# Rare copy number variation in autoimmune Addison’s disease

**DOI:** 10.3389/fimmu.2024.1374499

**Published:** 2024-03-18

**Authors:** Haydee Artaza, Daniel Eriksson, Ksenia Lavrichenko, Maribel Aranda-Guillén, Eirik Bratland, Marc Vaudel, Per Knappskog, Eystein S. Husebye, Sophie Bensing, Anette S. B. Wolff, Olle Kämpe, Ellen C. Røyrvik, Stefan Johansson

**Affiliations:** ^1^ Department of Clinical Science, University of Bergen, Bergen, Norway; ^2^ K. G. Jebsen Center for Autoimmune Diseases, University of Bergen, Bergen, Norway; ^3^ Department of Immunology, Genetics and Pathology, Uppsala University, Uppsala, Sweden; ^4^ Center for Molecular Medicine, Department of Medicine (Solna), Karolinska Institutet, Stockholm, Sweden; ^5^ Department of Medical Genetics, Oslo University Hospital, Oslo, Norway; ^6^ Department of Medical Genetics, Haukeland University Hospital, Bergen, Norway; ^7^ Mohn Center for Diabetes Precision Medicine, Department of Clinical Science, University of Bergen, Bergen, Norway; ^8^ Computational Biology Unit, Department of Informatics, University of Bergen, Bergen, Norway; ^9^ Department of Genetics and Bioinformatics, Health Data and Digitalization, Norwegian Institute of Public Health, Oslo, Norway; ^10^ Department of Medicine, Haukeland University Hospital, Bergen, Norway; ^11^ Department of Endocrinology, Karolinska University Hospital, Stockholm, Sweden; ^12^ Department of Molecular Medicine and Surgery, Karolinska Institutet, Stockholm, Sweden; ^13^ Department of Genetics and Bioinformatics, Norwegian Institute of Public Health, Bergen, Norway

**Keywords:** Addison’s disease, copy number variation, autoimmune, rare deletions, LRBA, BCL2L11

## Abstract

Autoimmune Addison’s disease (AAD) is a rare but life-threatening endocrine disorder caused by an autoimmune destruction of the adrenal cortex. A previous genome-wide association study (GWAS) has shown that common variants near immune-related genes, which mostly encode proteins participating in the immune response, affect the risk of developing this condition. However, little is known about the contribution of copy number variations (CNVs) to AAD susceptibility. We used the genome-wide genotyping data from Norwegian and Swedish individuals (1,182 cases and 3,810 controls) to investigate the putative role of CNVs in the AAD aetiology. Although the frequency of rare CNVs was similar between cases and controls, we observed that larger deletions (>1,000 kb) were more common among patients (OR = 4.23, 95% CI 1.85-9.66, p = 0.0002). Despite this, none of the large case-deletions were conclusively pathogenic, and the clinical presentation and an AAD-polygenic risk score were similar between cases with and without the large CNVs. Among deletions exclusive to individuals with AAD, we highlight two ultra-rare deletions in the genes *LRBA* and *BCL2L11*, which we speculate might have contributed to the polygenic risk in these carriers. In conclusion, rare CNVs do not appear to be a major cause of AAD but further studies are needed to ascertain the potential contribution of rare deletions to the polygenic load of AAD susceptibility.

## 1 Introduction

Autoimmune Addison’s disease (AAD) is a rare organ‐specific disease with a prevalence of approximately 100-200 per million in the western world (1). It is caused by an autoimmune destruction of the adrenal cortex, resulting in failure to produce glucocorticoid and mineralocorticoid hormones (1, 2). Fatigue, nausea, vomiting, abdominal pain, cramps, weight loss, skin hyperpigmentation and hypotension are typical symptoms of this disease (3). Diagnosis can be confirmed by the presence of autoantibodies against the adrenal enzyme 21-hydroxylase, and >90% of the patients have these markers at diagnosis (4, 5). A combination of environmental and genetic factors play an important role in this disorder, making it a complex task to reveal the actual underlying cause (1, 6). Previously, a single nucleotide polymorphism (SNP)-based GWAS showed that changes near multiple genes (ten associations at nine loci), most of them participating in the adaptive immune response, are associated with the risk of developing the condition (7). A considerable familial aggregation of AAD has been recognized for decades (8, 9) and twin studies have estimated the heritability to be approximately 0.97, 35-41% of this heritability is explained by common variants (7), indicating that genetic variation is a major contributor to disease liability (10). Taking these findings into consideration, a polygenic risk score (PRS) model was constructed and evaluated for AAD to estimate the risk of this condition at the collective influence of different genetic variants (11). This model showed a good performance for case-control differentiation and ability to estimate the risk of AAD at the individual level.

The term copy number variant (CNV) is typically used to describe DNA segments that vary in their copy number in the population and are referred to as deletions or duplications. These gains and losses may affect biological functions and disease susceptibility. Additionally, CNVs play an important role in Mendelian disorders, including hereditary cancer syndrome, cardiovascular, pediatric, neurological, neurodevelopmental, and neuropsychiatric disorders (12–14). Similarly, CNVs have been implicated in genetically complex diseases, particularly neuropsychiatric, neurodevelopmental, and neurodegenerative disorders (15–17). A role for CNVs in autoimmune and inflammatory phenotypes such as psoriasis, systemic lupus erythematosus, rheumatoid arthritis, Crohn’s disease, and type 2 diabetes, has also been suggested (12, 15–17). A previous CNV burden study in over 100,000 subjects of European ancestry has confirmed that rare CNVs are associated with autoimmune disorders (18). Despite the existing body of research on CNVs in human diseases, the knowledge of the role of CNVs in AAD has been limited by the very small sample sizes investigated (19). Here, we have conducted a genome-wide CNV analysis using SNP genotyping data from the largest Addison’s cohort collected so far (8, 9) to investigate the putative role of rare CNVs in the aetiology of AAD.

## 2 Materials and methods

### 2.1 Participants and ethical considerations

We recruited 1,526 participants with Addison’s disease from the Swedish and Norwegian Addison Registries, including 1,321 who fulfilled clinical diagnostic criteria for autoimmune primary adrenal insufficiency (7). Healthy controls (N = 4,471) were recruited from blood donor centers across Sweden and Norway. All participants were included as part of our previous Addison GWAS study, and details of sample treatment, extraction, genotyping and ethics, permissions are in the previous work (7) (Methods section).

All study subjects gave their informed consent and the studies were approved by the relevant local ethics committees (Sweden dnr 2008/296-31/2, Norway 2013-1504 and 2017-624).

### 2.2 Copy number variation calling and quality control

CNVs were identified from our previous AAD genotyping data (7). The intensity values from autosomal SNP probes, extracted from GenomeStudio software version 2.0.4, were used to detect genomic structural variations. A total of 692,367 SNPs markers from the Illumina Infinium Global Screen Array 1.0 were mapped to the GRCh37 assembly.

CNV calling was performed using PennCNV (20) version 1.0.5 with the default exclusion criteria. We removed outlier samples with respect to logR ratio standard deviation (LRR_SD < 0.3), B allele frequency drift (BAF_drift < 0.01) and waviness factor (|WF| < 0.05). An excessive number of CNVs associated with one sample (NumCNVs) could indicate a low quality sample (21), thus we visually inspected the distribution of NumCNVs across all samples and we defined our threshold for outlier exclusion as NumCNVs > 50. Furthermore, some genomic regions such as immunoglobulin, telomeric and centromeric regions are prone to accumulate spurious CNV calls; consequently, we removed all CNV calls overlapping at least 50% of these regions. The HLA region was retained despite its proneness to accumulate spurious CNV, as a strong association between the HLA region and autoimmune disease has been established (22).

PennCNV occasionally splits CNVs into several adjacent small fragments. Therefore, adjacent CNV calls fragments were merged into a single call. We merged adjacent CNVs when the fraction obtained by dividing the gap length between the two calls by the total length of the resulting merged call was <50%. We repeated the process once again by merging calls with a fraction <40% for best precision.

Only large CNVs, i.e. >50 kilobases (kb), covered by more than 5 probes, and belonging to samples with European ancestry (and only cases with serum autoantibodies against 21-hydroxylase), were included in our analysis.

We used PLINK (23) version 1.9 to evaluate the genetic relatedness, retaining samples with $\hat{\pi }\leq 0.1$ . According to these criteria, a total of 1,182 cases, 4,010 controls and 9,998 CNVs passed our filtering criteria and were taken forward for further analysis.

### 2.3 Defining rare CNVs

To derive a population-representative and array-specific set of common CNV-frequencies we estimated the population carrier frequency of CNVs by selecting a subset of samples from the total control group. From the controls, we extracted 200 random samples (25% Norwegian males, 25% Norwegian females, 25% Swedish males, and 25% Swedish females). We defined “common variants” as deletions or duplications with a carrier frequency >= 2% in this dataset and, subsequently, all CNVs overlapping at least 50% of their length with those variants were removed from the remaining controls and cases using Bedtools (24) v2.26.0. The 200 controls used for variant filtration were thereafter excluded from further analyses.

### 2.4 Burden analysis

First we assessed the aggregated frequency for all rare deletions and duplications in cases versus controls. Then we split CNVs, both deletions and duplications, into five length intervals: 50-100, 100-200, 200-500, 500-1,000 kb, and >1,000 kb. The CNV frequency in cases and controls was estimated by the number of variants in each length interval divided by the total number of cases or controls, respectively. Two proportion test and odds ratio estimation were used to test for differences in CNV frequencies between patients and controls using R version 3.4.4, specifically the *stats* and *fmsb* packages, respectively. The overall frequencies of singleton CNVs (CNV that appears only in one individual) were assessed using the same procedure. Following Bonferroni adjustment for multiple testing correction we employed a significance threshold of p = 0.005 (10 tests).

We also confirmed that the results were consistent across the two study cohorts by performing the analyses stratified by Norwegian and Swedish ancestries.

### 2.5 Rare CNVs enrichment analysis

We performed gene-set enrichment analyses for CNV data as implemented in Plink v.1.07 which is robust to case-control differences in CNV size, CNV rate, and systematic differences in gene size (25). We evaluated: (a) general gene enrichment among case CNVs (–cnv-count, –cnv-enrichment-test), and (b) candidate gene enrichment (enrichment of pathway genes) relative to all genes (–cnv-count, –cnv-subset, –cnv-enrichment-test). In (b) we used two genes lists: one from Genomics England PanelApp (26) including primary immunodeficiency and congenital adrenal hypoplasia panels (536 genes in total, **Supplementary Table A**, panelApp_AI_genes_inhe_pattern); the second list is an in-house curated gene list, containing genes based on their known role in basic immunity, inflammation and autoimmune disease (27) (1,846 genes in total, **Supplementary Table A**, Curated_AI_genes). The gene annotation for the entire genome (generated from UCSC Table Browser build hg19/GRCh37), was downloaded from the Plink resources page (28). Enrichment was considered significant at the level of p < 0.008 (Bonferroni adjustment for two gene-lists and all genes, each for deletions and duplications).

All rare large CNVs (>1 Mb) and rare CNVs overlapping the Genomics PanelApp immunity list were evaluated for putative pathogenicity in accordance with standard clinical diagnostics/ACMG guidelines (29) and assessed for syndromic forms of immunity related disease. Furthermore, exploratory evaluations were performed to search for putative overlap with risk variants implicated in more common forms of autoimmune disease using the following databases: Database of Genomic Variations (DGV) (30); DatabasE of genomiC varIation and Phenotype in Humans using Ensembl Resources (DECIPHER) (31); Online Mendelian Inheritance in Man (OMIM) (32); the GWAS-catalog (33), and literature searches.

### 2.6 Polygenic risk score in carriers of rare large deletions

The PRS model was developed from the original case-control GWAS-study of AAD and healthy controls (7, 11). A total of 1,182 cases and 4,010 controls, all of them unrelated and European-ancestry samples passing the quality control, were selected for the PRS estimation. We defined four CNV-carrier groups to assess their AAD-PRS distribution: Large rare CNV carrier cases (n = 13), non-carriers cases (n = 1,169), carrier controls (n = 10) and non-carriers controls (n = 4,000). Due to slight differences in the quality control process to select individuals between the original GWAS-study and the CNV-study, a few non-carriers were not included in the PRS model (2 cases and 6 controls). However, as none of them are AAD patients with deletions, this slight difference should have a negligible impact on our study. Consequently, we tested carrier cases (n = 13), non-carriers cases (n = 1,167), carrier controls (n = 10) and non-carriers controls (n = 3,994). The PRS is expressed as a Z-score with mean 0 and standard deviation 1 in healthy controls. Groupwise comparisons were made using Wilcoxon rank sum test with continuity correction.

## 3 Results

**Figure 1:**
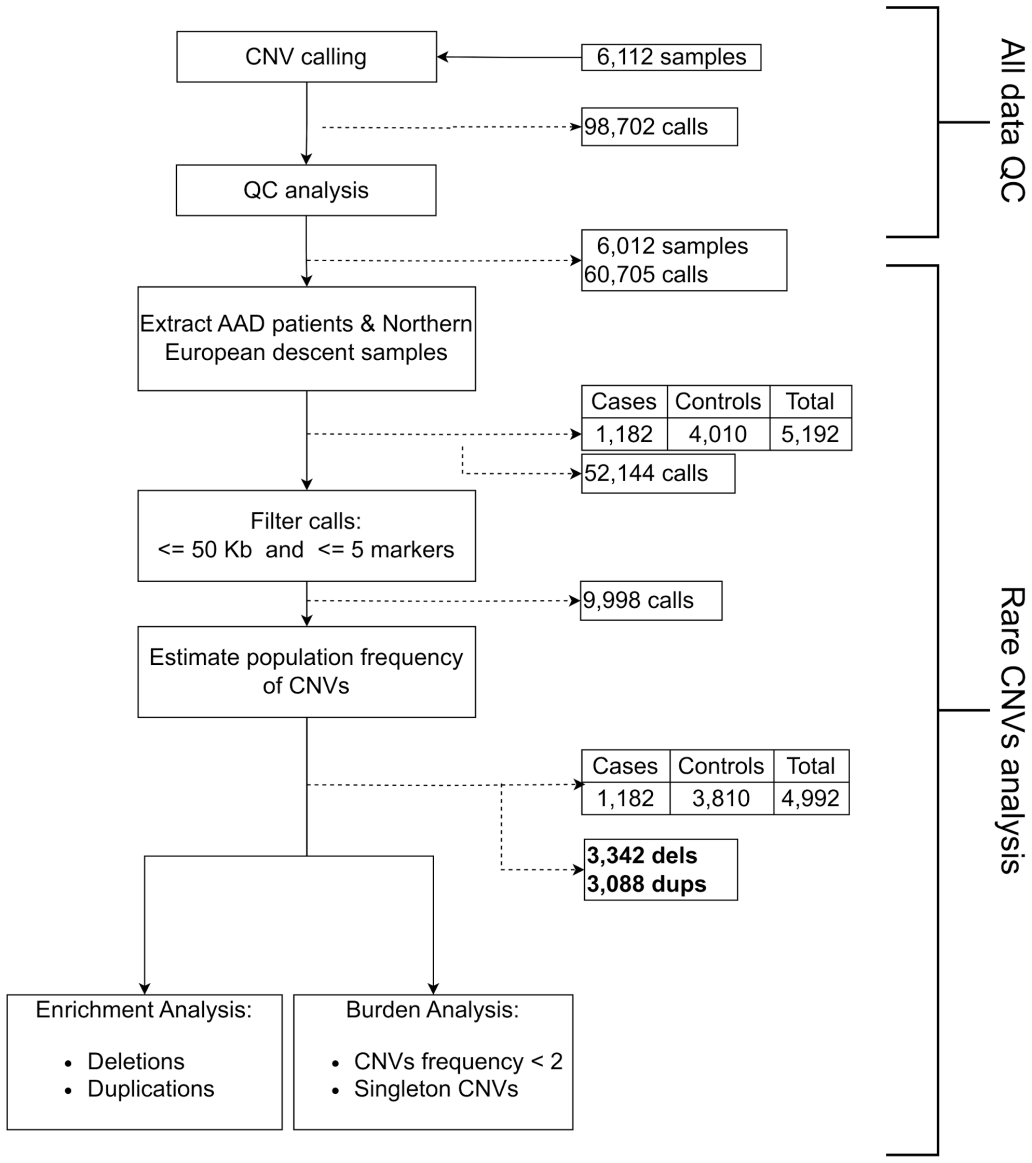
Flow chart of the individuals and calls included in our CNV analysis.

CNV calling was performed in 1,526 cases and 4,471 controls genotyped with the Illumina Infinium Global Screen Array 1.0 using 692,367 markers, followed by a strict quality control pipeline (see Methods section). Only cases with serum autoantibodies against 21-hydroxylase were taken forward for analysis and samples of non-European descent were excluded. This yielded a total sample number of 1,182 cases and 4,010 controls for further analyses (**Figure 1**). CNV frequencies were similar in the Norwegian and Swedish sample sets (**Supplementary Tables 1 and 2**). The primary analyses were restricted to rare CNVs of >50 kb and with >5 probes, and estimated to be present in less than 2% of individuals (corresponding to a 1% allele frequency) in a randomly extracted subset of 200 controls. These control samples were subsequently removed from the study (Materials and Methods). This resulted in a total of 3,342 rare deletions and 3,088 rare duplications among the 1,182 cases and 3,810 controls that passed QC (**Figure 1**). There were no apparent differences in the cumulative distribution of rare deletions in patients compared to controls (OR = 1.06, 95% CI 0.92-1.23, p = 0.39) or for duplications (OR = 0.95, 95% CI 0.83-1.09, p = 0.49) (**Table 1**). However, when binning the CNVs according to their size, a higher frequency for the longest rare deletions (>1,000 kb) was observed among cases (n = 13/1182) compared to controls (n = 10/3810) (OR = 4.23, 95% CI 1.85-9.66, p = 0.0002) (**Table 2**). Results were similar when restricting the analysis to singleton CNVs, with a higher frequency of the largest singleton deletions (>1,000 kb) among cases compared to controls (OR = 6.48, 95% CI 1.95-21.57, p = 0.0005) (**Supplementary Table 3**), and we also observed a trend for higher cumulative frequency of singleton deletions and duplications in cases compared to controls (**Supplementary Table 4**).

**Table 1:** Overall rare deletion and duplication frequency distribution.

CNV type	Counts		Frequency		Association	
	CNVs Cases [n = 1182]	CNVs Controls [n = 3810]	Cases	Controls	OR (95% CI)	*P*
DELs	827	2615	0.70	0.69	1.06 (0.92-1.23)	0.39
DUPs	721	2367	0.61	0.62	0.95 (0.83-1.09)	0.49

**Table 2:** Rare CNV frequency distribution by interval size in cases vs. controls.

CNV type	CNVs length	Counts		Frequency		Association	
				Cases	Controls	OR (95% CI)	*P*
		CNVs Cases [1182]	CNVs Controls [3810]				
DELs	50KB_100KB	435	1298	0.37	0.34	1.13 (0.98-1.29)	0.09
	100KB_200KB	260	919	0.22	0.24	0.89 (0.76-1.04)	0.13
	200KB_500KB	102	323	0.09	0.09	1.02 (0.81-1.29)	0.87
	500KB_1000KB	17	65	0.01	0.02	0.84 (0.49-1.44)	0.53
	> 1000KB	13	10	0.011	0.003	4.23 (1.85-9.66)	0.0002
DUPs	50KB_100KB	297	1050	0.25	0.28	0.88 (0.76-1.02)	0.10
	100KB_200KB	204	614	0.17	0.16	1.09 (0.91-1.29)	0.35
	200KB_500KB	157	488	0.13	0.13	1.04 (0.86-1.26)	0.67
	500KB_1000KB	48	150	0.04	0.04	1.03 (0.74-1.44)	0.85
	> 1000KB	15	65	0.013	0.017	0.74 (0.42-1.30)	0.30

Although patients negative for serum autoantibodies against 21-hydroxylase were excluded from the primary study, we explored the CNV distribution in this group (201 individuals). These patients presented similar CNV frequencies to both controls and 21-hydroxylase autoantibody positive patients. A further CNV breakdown according to size did not indicate any gross differences, however the very low number of individuals in this group limits the power substantially (**Supplementary Table 8**).

The apparent excess of long, rare deletions (>1,000 kb) in 21-hydroxylase autoantibody positive cases (n = 13 patients, OR = 4.23, 95% CI 1.85-9.66, p < 0.0002) prompted us to further investigate their putative role in AAD-susceptibility. These deletions were equally distributed between Norwegian and Swedish patients (7 Norwegians and 6 Swedes). None of the deletions overlap with known syndromic regions or genes implicated in Mendelian immune-related disorders, and none qualify as likely pathogenic or pathogenic according to American College of Medical Genetics and Genomics (ACMG) guidelines (34). We also surveyed the case-deletions against polygenic signals from the GWAS-catalog (**Table 3**). A deletion on chromosome 2q13 in one patient (underlined row 3 in **Table 3**) covers regions of GWAS-association to vitiligo (35) (near *MIR4435-2HG*), alopecia areata (36) (near *ACOXL*), multiple sclerosis (37) (near *MERTK*), systemic lupus erythematosus (38) (near *BCL2L11, ACOXL*, and *MIR4435-2HG*) and type 1 diabetes (39) (near *ACOXL*). The deletion is also known as the recurrent 2q13 microdeletion associated with variable penetrance and phenotypic expression including developmental delay, congenital heart disease and autism, but is also present in apparently healthy carriers (40). These previous publications have not reported immunological comorbidities in their clinical descriptions. Another two patients (underlined row 11 and 16 in **Table 3**) showed deletions encompassing genes associated with lymphocyte count (41–43) (*RBPMS-AS1, PMP22* and *TEKT3*, respectively). It should be mentioned that a deletion encompassing a GWAS hit for type 1 diabetes was also found in controls (**Table 3**).

**Table 3:** Large (>1000kb) rare deletions.

Chr:bp-bp	Mb	Cat=N	Immune phenotype	Mapped genes	GWAS Ref.
2:25185405-26797358	1.61	U=1	1. Type 1 diabetes 2. Lymphocyte counts	EFR3B DNMT3A	1. Study GCST001255 (Bradfield, J. P. et al., 2011) 2. Study GCST004627 (Astle, W. J. et al., 2016)
2:106874835-108440432	1.57	U=1	1.Serum immune	EEF1A1P12, ST6GAL2	1. Study GCST010146 (Zhang, R. et al., 2020).
2:111399346-113093928	1.69	A=1	1.Vitiligo 2. Alopecia areata 3. Multiple Sclerosis 4. Systemic Lupus Erythematosus 5. Type 1 diabetes	MIR4435-2HG ACOXL MERTK BCL2L11, MIR4435-2HG ACOXL	1. Study GCST004785 (Jin, Y. et al., 2016) 2. Study GCST004866 (Betz, R. C. et al., 2015) 3. Study GCST009597 (International Multiple Sclerosis Genetics Consortium, 2019) 4. Study GCST011956 (Yin, X. et al., 2021) 5. Study GCST005536 (Onengut-Gumuscu, S. et al., 2015)
2:122785624-125774467	2.56	A=1 U=1	None		
3:1632353-2788576	1.16	U=1	1. Serum immune biomarker levels	CNTN4	1. Study GCST010146 (Zhang, R. et al., 2020)
3:144445174-145758050	1.31	A=1	None		
3:174910449-176153637	1.24	U=1	None		
4:188917331-190459134	1.54	U=1	None		
5:24837197-26174342	1.34	A=1	None		
5:19293784-20970950	1.68	A=1	None		
8:28935001-30828160	1.89	A=1	1. Lymphocyte count	RBPMS-AS1, TUBBP1	1. Study GCST90002320 (Chen, M.-H. et al., 2020); and study GCST90002388 (Vuckovic, D. et al., 2020)
13:56798769-57813567	1.01	A=1 U=1	None		
13:55968185-57432964	1.46	A=1	None		
13:80959100-82030660	1.07	A=1	None		
16:15125441-16305355	1.18	A=1 U=2	1. Serum immune biomarker levels	NDE1, MYH11	1. Study GCST010146 (Zhang, R. et al., 2020)
17:14098277-15430857	1.31	A=2	1. Lymphocyte count	PMP22, TEKT3	1. Study GCST011881 (1. Okada, D. et al., 2021)
18:63697503-66099958	2.40	A=1	None		
21:22668119-25713704	3.05	U=1	None		

Chr:bp-bp - deletion coordinates; GRCh37; Mb - length of deletion in megabases. Cat=N - category in which deletion found; and number in each category; A = affected with Addison’s; U = unaffected; Immune phenotype - reported immune-related phenotype for which there is a genome-wide significantly associated locus falling within the deletion (source: GWAS catalog); Mapped genes - Gene(s) mapped to the strongest SNP as listed in GWAs catalog. If the SNP is located within a gene; that gene is listed. If the SNP is located within multiple genes; these genes are listed separated by commas. If the SNP is intergenic; the upstream and downstream genes are listed; separated by a hyphen (from GWAS Catalog documentation); GWAS Ref. - reference to the relevant GWAS. Deletion with majority overlapping coordinates have been merged; to cover their cumulative greatest extent.

Underline words means Case-deletions covering regions of GWAS-association to immune diseases.

**Figure 2:**
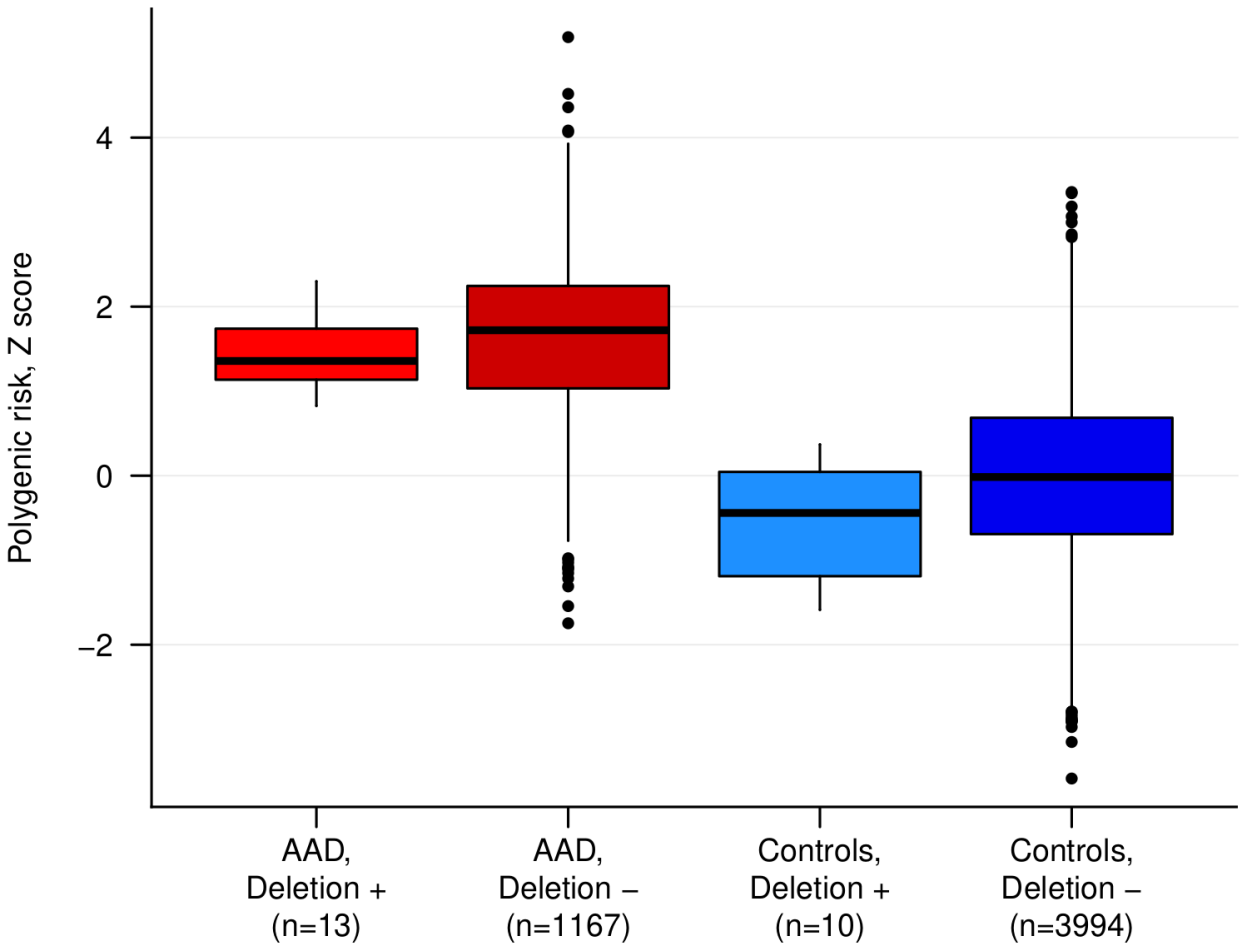
The polygenic risk score (PRS) for autoimmune Addison’s disease (AAD) in cases and controls stratified by rare large deletion carrier status. The difference between cases with and without a deletion was not statistically significant. ‘+’ indicates carriers, and ‘-’ indicates non-carriers.

We used a recent PRS model for AAD (11) (PRS14_AAD_) to test whether the PRS-distribution in patients with rare large deletions (>1,000 kb) is different compared to patients without rare large deletions, which can indicate different etiologies. For this purpose, we compared the previously derived AAD-PRS for carrier patients (n = 13), non-carriers patients (n = 1,167), carrier controls (n = 10) and non-carrier controls (n = 3,994) (**Figure 2**). Median PRS in carrier patients with AAD was similar to that of non-CNV-carrier AAD-patients (p = 0.30) and significantly higher than in normal controls (p = 1.7x10^-6^) (**Figure 2**). Furthermore, all large rare deletion carriers had a PRS within the range of non-carrier AAD-cases, and all scores were above the 75:th percentile of control subjects. We also compared the clinical presentation, age-of onset and comorbidity profile for this group, and found no substantial clinical differences compared to the other AAD-patients (**Supplementary Figure 1** and **Supplementary Table 7**). Taken together, these analyses suggest that the putative enrichment of large rare CNVs identified among AAD-cases here is not due to the existence of as-of-yet non-identified Mendelian syndromes, but possibly may implicate a rare-variant contribution to the polygenic load in some of the carriers.

We next investigated whether particular gene-sets were enriched for rare CNVs among cases. We evaluated two candidate gene-set lists: the first list is of genes that are included in the primary immunodeficiency and congenital adrenal hypoplasia panels from the Genomics England PanelApp (https://panelapp.genomicsengland.co.uk/); the second list is an in-house curated list of genes based on their known role in basic immunity, inflammation, and autoimmune disease. Results did not reveal any evidence for an overall enrichment of rare CNVs overlapping with immune related genes among cases (**Supplementary Table 5**).

In an attempt to identify single rare high-penetrance CNVs, we also surveyed each rare deletion overlapping the immune-panel genes listed in **Supplementary Table 6**. None of the CNVs was evaluated as pathogenic or likely pathogenic using the ACMG guidelines for pathogenicity and none of the CNVs were enriched in cases compared to controls, albeit it must be noted that power is very low at the single CNV level.

## 4 Discussion

Our investigation presents the largest CNV analysis in individuals with AAD to date. Although we did not detect an overall significant CNV frequency difference between cases and controls, we did find some evidence of enrichment of large deletions (>1,000 kb) in AAD patients. Polygenic risk scores for AAD were similarly high for carrier and non-carrier patients indicating shared etiology. It is therefore unlikely that these rare CNVs constitute high-penetrance Mendelian variants, but rather suggest that they might contribute to the polygenic susceptibility in carrier individuals. Future studies examining this question are needed.

There are some interesting results among the rare CNV deletions. We restrict the discussion of our findings to a few specific deletions that we consider to have the highest probability of being of functional relevance to the development of AAD, namely those that contain loci associated with other autoimmune diseases or immuno-related phenotypes and that are exclusively found in patients. While we cannot claim any statistical evidence for the involvement of these CNVs, restricted as they are to the rare category, we highlight several examples that could plausibly be considered additional risk variants to the common SNPs identified in Eriksson et al. (7).

Functionally the most prominent of these is the 1.7 Mb deletion on chromosome 2 (**Table 3**), also known as the rare recurrent 2q13 microdeletion that has been linked to a range of cognitive conditions, albeit often inherited from non-affected parents (40). This region has SNP-based associations with a wide range of autoimmune diseases (vitiligo, alopecia areata, multiple sclerosis, systemic lupus erythematosus, and type 1 diabetes), and contains *BCL2L11*, encoding BIM. BIM is a proapoptotic protein, which has a very specific thymic function, studied in murine systems (44). It is required for negative selection of thymocytes reacting to tissue-restricted antigens (TRAs) (45, 46). Autoimmune Regulator (AIRE) is needed to induce the expression of these TRAs in the thymic medulla, and mutations in AIRE cause the monogenic syndrome APS-1, of which AAD is a major constituent (3, 47). Two variants at the *AIRE* locus were among the strongest identified in the AAD GWAS (7) and included the low frequency coding variant p.R471C (2% population allele frequency) associated with a 3.4 fold increased odds of developing AAD. While BIM deficiency alone seems insufficient to produce a break in central tolerance and consequent autoimmunity, in concert with deficiency of PUMA (another pro-apoptotic protein) such a phenotype emerges in mouse models of immunity (46, 48). It is therefore possible that a single-copy deletion of *BCL2L11* may be only very mildly deleterious, but in concert with other risk variants involved in clonal deletion to TRAs it could promote an autoimmune phenotype. In our case, the patient carrying this deletion is heterozygous for both the aforementioned *AIRE* risk alleles.

It is also notable that we find heterozygous *LRBA* deletions (**Supplementary Table 6**) in two patients, one Swedish and one Norwegian, and in no controls. Deletions in this gene are extremely rare. The deletion in the Swedish patient covers the 5’ end of the gene, inclusive of promoter, and the deletion in the Norwegian patient deletes exons 3 and 4 of the gene and thus both are expected to lead to only a single functional copy in the affected patients. LRBA regulates CTLA4 levels, likely by promoting its recycling to the cell surface over degradation (49). CTLA4 is constitutively expressed on regulatory T cells (Tregs), is crucial to their suppressive capabilities, and is a recurrently associated locus with autoimmune diseases, including AAD (2). Patients with LRBA deficiency due to bi-allelic loss of function mutations display significant immune dysregulation (50), but Tregs from patients with heterozygous *LRBA* mutations also show loss of CTLA4 expression, though less so than homozygous ones (49). Both *LRBA* hemizygous AAD patients carry the common AAD *CTLA4* risk allele, in either the homozygous or heterozygous state, and given that the postulated *CTLA4* risk allele effect is related to gene expression levels (7) it is possible that the loss of a copy of *LRBA* would compound the CTLA4-based autoimmunity risk. Both patients carrying *LRBA* deletions received an AAD diagnosis quite young (19 and 16 years, putting them in the lowest 16% of the age-of-diagnosis range), where the median age-at-diagnosis for the patient cohort is 33 years (mean 35).

As both of our highlighted, ultra-rare, patient-specific CNVs happen to encompass genes whose products participate in the same molecular pathways as common risk variants genes, and in our case present with such common risk variants, it is difficult to speculate as to whether or not the CNVs might exert an independent effect on disease risk or not.

The natural complexity of CNVs and their role in increasing immunological diversity (51) make the CNV analysis challenging. Although our population-based study is one of the largest for a rare disease such as AAD (prevalence = 0.02%), the power limits us to qualitative assessments of the ultra rare variants discovered. We performed a rigorous quality control to avoid any technical artifacts related to sample quality, genetic structure in our two populations, and possible frequency bias. Therefore, we do not expect that our results can be attributed to batch effects or low quality data.

In conclusion, rare CNVs do not appear to have a major role in AAD predisposition but our results suggest that rare deletions may contribute to the general polygenic risk for the disease. This hypothesis should remain under consideration until larger studies have been performed.

## Data availability statement

The datasets presented in this article are not readily available because of restrictions related to ethical approvals regarding sharing of human genomic data. Requests to access the datasets should be directed to AW (Anette.boe@uib.no). Access to the dataset requires an ethical approval from the requesting party.

## Ethics statement

The studies involving humans were approved by the local ethics committees in Stockholm, Sweden (dnr 2008/296-31/2), and Western Norway (biobank 2013-1504, project 2017-624). The studies were conducted in accordance with the local legislation and institutional requirements. The participants provided their written informed consent to participate in this study. Written informed consent was obtained from the individual(s) for the publication of any potentially identifiable images or data included in this article.

## Author contributions

HA: Formal Analysis, Investigation, Visualization, Writing – original draft, Writing – review & editing, Data curation. DE: Formal Analysis, Visualization, Writing – review & editing, Data curation, Investigation. KL: Writing – review & editing, Investigation, Visualization. MA-G: Writing – review & editing, Investigation. EB: Writing – review & editing, Investigation. MV: Writing – review & editing, Investigation. PK: Writing – review & editing, Investigation. EH: Funding acquisition, Writing – review & editing, Investigation. SB: Writing – review & editing, Investigation. AW: Supervision, Writing – review & editing, Investigation. OK: Writing – review & editing, Funding acquisition, Investigation. ER: Conceptualization, Supervision, Writing – review & editing, Formal Analysis, Investigation, Writing – original draft. SJ: Conceptualization, Supervision, Writing – review & editing, Formal Analysis, Funding acquisition, Investigation, Project administration, Writing – original draft.
