# Supplementary material for: Rare copy number variation in autoimmune Addison’s disease
Source: Front Immunol. 2024 Mar 18;15:1374499. doi: 10.3389/fimmu.2024.1374499 (PMC10982488; doi:10.3389/fimmu.2024.1374499)
Supplement: Supplementary file 2 [file Image_1.pdf]

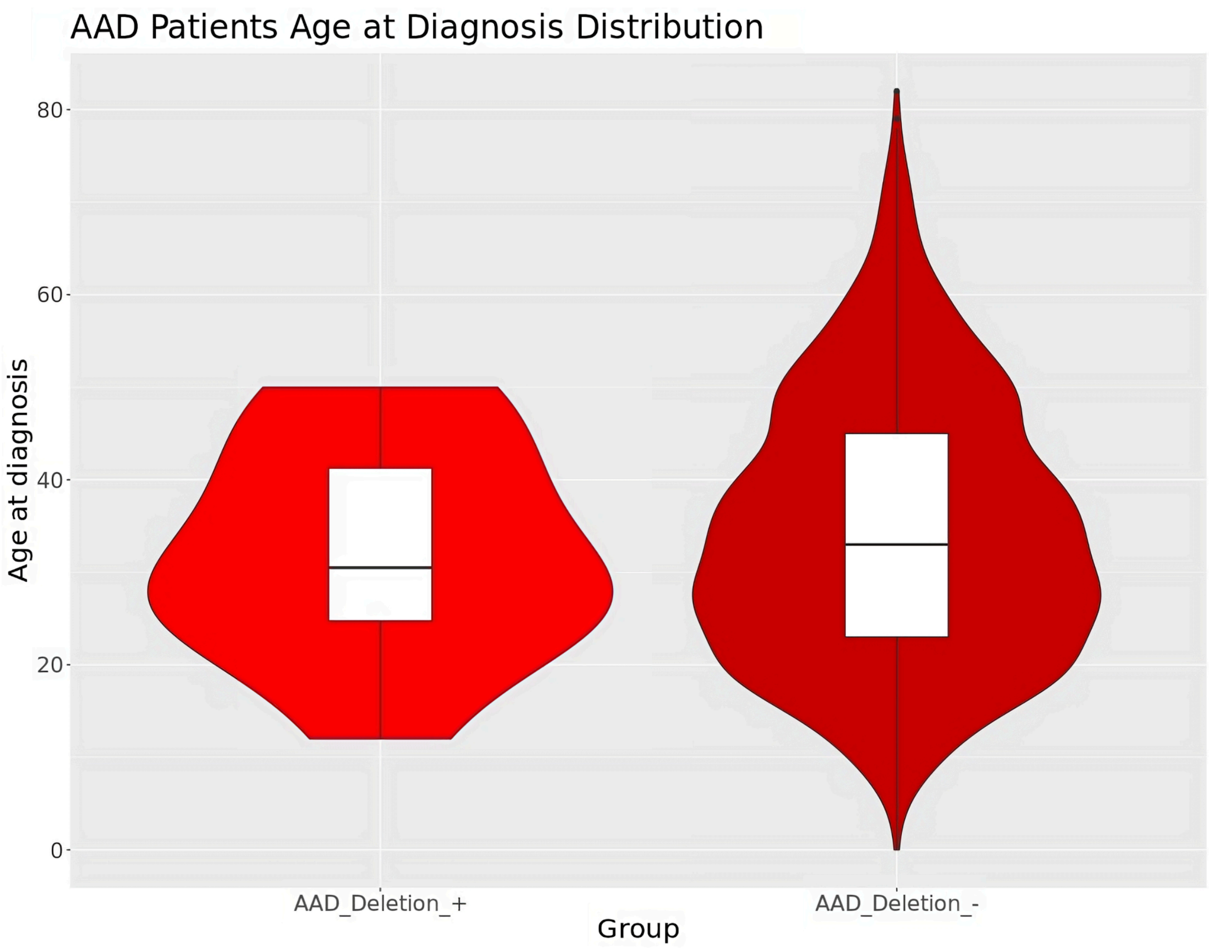

Supplementary Figure 1. Violin Plot of the age at diagnosis distribution of AAD patients with (AAD_Deletion_+) and without (AAD_Deletion_-) large rare deletions (>1000 kb). The average age of carriers is 30.50 (IQR: [24.75-41.25], Min-Max Range: [12-50]), and 33 for non-carriers ((QR: [23-45], Min-Max Range: [0-82]). No significant difference was found.
