# Supplementary material for: Rare copy number variation in autoimmune Addison’s disease
Source: Front Immunol. 2024 Mar 18;15:1374499. doi: 10.3389/fimmu.2024.1374499 (PMC10982488; doi:10.3389/fimmu.2024.1374499)
Supplement: Supplementary file 3 [file Table_1.pdf]

Supplementary Table 1. Rare CNV frequency distribution by nationality

CNV type	CNVs length	Cases CNVs Counts		Control CNVs Counts		Cases Frequency			Controls Frequency		
		NOR[1] [540]	SWD[2] [642]	NOR [1718]	SWD [2092]	NOR	SWD	*P**	NOR	SWD	*P**
DELs	50KB_100KB	199	236	589	709	0.37	0.37	1.00	0.34	0.34	0.83
	100KB_200KB	121	139	430	489	0.22	0.22	0.81	0.25	0.23	0.25
	200KB_500KB	50	52	148	175	0.09	0.08	0.55	0.09	0.08	0.83
	500KB_1000KB	11	6	33	32	0.02	0.01	0.18	0.02	0.02	0.42
	1000KB_>	7	6	4	6	0.01	0.01	0.75	0.00	0.00	1.00
DUPs	50KB_100KB	148	149	456	594	0.27	0.23	0.11	0.27	0.28	0.22
	100KB_200KB	77	127	253	361	0.14	0.20	0.02	0.15	0.17	0.04
	200KB_500KB	77	80	216	272	0.14	0.12	0.41	0.13	0.13	0.73
	500KB_1000KB	21	27	83	67	0.04	0.04	0.90	0.05	0.03	0.01
	1000KB_>	6	9	26	39	0.01	0.01	0.85	0.02	0.02	0.48

(1) Norwegian, (2) Swedish

(*) Two proportion test used to compare two observed proportions. *P* (p-value of the test): testing the null that the proportions of CNVs (deletions and duplications) in Norwegians and Swedish are the same.
