# Supplementary material for: Rare copy number variation in autoimmune Addison’s disease
Source: Front Immunol. 2024 Mar 18;15:1374499. doi: 10.3389/fimmu.2024.1374499 (PMC10982488; doi:10.3389/fimmu.2024.1374499)
Supplement: Supplementary file 4 [file Table_2.pdf]

Supplementary Table 2. Singleton CNVs frequency distribution by nationality

CNV type	CNVs length	Cases CNVs Counts		Control CNVs Counts		Cases Frequency			Controls Frequency		
		NOR[1] [540]	SWD[2] [642]	NOR [1718]	SWD [2092]	NOR	SWD	*P**	NOR	SWD	*P**
DELs	50KB_100KB	53	62	112	184	0.10	0.10	1.00	0.07	0.09	0.01
	100KB_200KB	29	34	69	117	0.05	0.05	1.00	0.04	0.06	0.03
	200KB_500KB	10	23	45	47	0.02	0.04	0.10	0.03	0.02	0.52
	500KB_1000KB	2	3	16	9	0.004	0.005	1.00	0.009	0.004	0.09
	1000KB_>	6	2	1	3	0.011	0.003	0.19	0.001	0.001	0.76
DUPs	50KB_100KB	46	42	99	165	0.09	0.07	0.24	0.06	0.08	0.01
	100KB_200KB	23	41	55	76	0.04	0.06	0.14	0.03	0.04	0.52
	200KB_500KB	18	31	49	80	0.03	0.05	0.26	0.03	0.04	0.12
	500KB_1000KB	11	20	27	35	0.02	0.03	0.33	0.02	0.02	0.91
	1000KB_>	1	6	11	26	0.002	0.009	0.20	0.01	0.01	0.09

(1) Norwegian, (2)Swedish

(*) Two proportion test used to compare two observed proportions. P (p-value of the test): testing the null that the proportions of CNVs (deletions and duplications) in Norwegians and Swedish are the same.
