# Supplementary material for: Rare copy number variation in autoimmune Addison’s disease
Source: Front Immunol. 2024 Mar 18;15:1374499. doi: 10.3389/fimmu.2024.1374499 (PMC10982488; doi:10.3389/fimmu.2024.1374499)
Supplement: Supplementary file 5 [file Table_3.pdf]

**Supplementary Table 3.** Singleton CNVs frequency distribution by interval size in cases vs. controls

| CNV type | CNVs length | Counts | | Frequency | | Association | |
|---|---|---|---|---|---|---|---|
| | | CNVs Cases [1182] | CNVs Controls [3810] | Cases | Controls | OR (95% CI) | *P* |
| DELs | 50KB_100KB | 115 | 296 | 0.10 | 0.08 | 1.28 (1.02-1.60) | 0.03 |
| | 100KB_200KB | 63 | 186 | 0.05 | 0.05 | 1.10 (0.82-1.47) | 0.54 |
| | 200KB_500KB | 33 | 92 | 0.03 | 0.02 | 1.16 (0.78-1.74) | 0.47 |
| | 500KB_1000KB | 5 | 25 | 0.00 | 0.01 | 0.64 (0.25-1.68) | 0.37 |
| | > 1000KB | 8 | 4 | 0.007 | 0.001 | 6.48 (1.95-21.57) | 0.0005 |
| DUPs | 50KB_100KB | 88 | 264 | 0.07 | 0.07 | 1.08 (0.84-1.39) | 0.55 |
| | 100KB_200KB | 64 | 131 | 0.05 | 0.03 | 1.61 (1.18-2.18) | 0.002 |
| | 200KB_500KB | 49 | 129 | 0.04 | 0.03 | 1.23 (0.88-1.73) | 0.22 |
| | 500KB_1000KB | 31 | 62 | 0.03 | 0.02 | 1.63 (1.05-2.52) | 0.03 |
| | > 1000KB | 7 | 37 | 0.006 | 0.010 | 0.61 (0.27-1.37) | 0.22 |
