# Supplementary material for: Rare copy number variation in autoimmune Addison’s disease
Source: Front Immunol. 2024 Mar 18;15:1374499. doi: 10.3389/fimmu.2024.1374499 (PMC10982488; doi:10.3389/fimmu.2024.1374499)
Supplement: Supplementary file 6 [file Table_4.pdf]

Supplementary Table 4. Overall singleton deletions and duplications frequency distribution

	Counts		Frequency		Association	
	**CNVs Cases [n = 1182]**	**CNVs Controls [n = 3810]**	**Cases**	**Controls**	**OR (95% CI)**	***P***
DELs	224	603	0.19	0.16	1.24 (1.05-1.47)	0.01
DUPs	239	623	0.20	0.16	1.30 (1.10-1.53)	0.002
