# Supplementary material for: Rare copy number variation in autoimmune Addison’s disease
Source: Front Immunol. 2024 Mar 18;15:1374499. doi: 10.3389/fimmu.2024.1374499 (PMC10982488; doi:10.3389/fimmu.2024.1374499)
Supplement: Supplementary file 7 [file Table_5.pdf]

Supplementary Table 5. Enrichment plink test results

(a)	**Genic CNVs (genes in hg19-Genome-Assembly)**			
DELs	**TEST**	**BETA**	**OR**	***P***
	GCNT	0.015	1.02	0.401
	NSEG	-0.020	0.98	0.787
	AVGKB	3.11E-04	1.00	0.321
DUPs	GCNT	-0.002	1.00	0.846
	NSEG	-0.030	0.97	0.705
	AVGKB	4.91E-05	1.00	0.796
**(b)**	**Pathway genes in PanelApp gene-setlist (Supplementary Table A)**			
DELs	**TEST**	**BETA**	**OR**	***P***
	GCNT	0.208	1.23	0.069
	NSEG	-0.016	0.98	0.824
	AVGKB	3.98E-04	1.00	0.186
DUPs	GCNT	-0.221	0.80	0.241
	NSEG	-0.026	0.97	0.731
	AVGKB	5.26E-05	1.00	0.751
**(c)**	**pathway genes in curated gene-setlist (Supplementary Table A)**			
DELs	**TEST**	**BETA**	**OR**	***P***
	GCNT	0.087	1.09	0.277
	NSEG	-0.009	0.99	0.899
	AVGKB	3.57E-04	1.00	0.237
DUPs	GCNT	0.001	1.00	0.986
	NSEG	-0.036	0.96	0.638
	AVGKB	2.96E-05	1.00	0.862

Logistic regression parameters: GCNT (Gene counts), NSEG (Number of segments or CNVs), and AVGKB (Average size of CNVs).
