# Supplementary material for: Rare copy number variation in autoimmune Addison’s disease
Source: Front Immunol. 2024 Mar 18;15:1374499. doi: 10.3389/fimmu.2024.1374499 (PMC10982488; doi:10.3389/fimmu.2024.1374499)
Supplement: Supplementary file 8 [file Table_6.pdf]

Supplementary Table 6. Genes (33 in total) from PanelApp list spanned by rare deletions (280 in total). Column Cases and Controls show the number of deletions in each spanning gene.

Del Chr:Position (% overlapping)	Gene	Dels Cases	Dels Controls	OR (95%CI)	*P**	Inheritance
1:92309464-92941657 (11%)	GFI1	1	0	9.51 (0.39-233.80)	0.091	MONOALLELIC, autosomal or pseudoautosomal, NOT imprinted
1:119202269-120138931 (100%)	HSD3B2	0	1	1.06 (0.04-25.92)	0.974	BIALLELIC, autosomal or pseudoautosomal
1:145383239-145738979 (100%)	POLR3C	0	3	0.45 (0.02-8.75)	0.589	MONOALLELIC, autosomal or pseudoautosomal, NOT imprinted
1:149920593-150058019 (24%)	VPS45	1	0	9.51 (0.387-23.80)	0.091	BIALLELIC, autosomal or pseudoautosomal
1:196733306-196799813 (0%) 16.7 Kb downstream	CFH	1	2	1.58 (0.14-17.50)	0.705	BOTH monoallelic and biallelic, autosomal or pseudoautosomal
1:196733306-196799813 (74%)	CFHR3	8	24	1.06 (0.47-2.36)	0.895	BOTH monoallelic and biallelic, autosomal or pseudoautosomal
1:196748972-196821486 (100%)	CFHR1	17	42	1.23 (0.73-2.28)	0.382	BOTH monoallelic and biallelic, autosomal or pseudoautosomal
1:196797198-196887480 (99%)	CFHR4	20	54	1.18 (0.70-1.99)	0.537	BOTH monoallelic and biallelic, autosomal or pseudoautosomal
2:25185405-26797358 (100%)	POMC	0	1	1.06 (0.04-25.92)	0.974	-

2:219883772-219965682 (30%)	NHEJ1	1	0	9.51 (0.39-233.80)	0.091	BIALLELIC, autosomal or pseudoautosomal
2:230959540-231022596 (0%)<br>11.0 Kb upstream	SP110	0	1	1.06 (0.04-25.92)	0.974	BIALLELIC, autosomal or pseudoautosomal
2:233215403-233301535 (0%)<br>5.3 Kb upstream	ALPI	2	0	15.88 (0.76-331.23)	0.016	BIALLELIC, autosomal or pseudoautosomal
3:48540149-48645842 (100%)	COL7A1	0	1	1.06 (0.04-25.92)	0.974	BOTH monoallelic and biallelic, autosomal or pseudoautosomal
3:195794420-195844938 (44%)	TFRC	0	1	1.06 (0.043-25.92)	0.974	BIALLELIC, autosomal or pseudoautosomal
4:123353432-124157453 (100%)	IL21	0	1	1.06 (0.043-25.92)	0.974	BIALLELIC, autosomal or pseudoautosomal
4:151790540-151961655 (19%)	LRBA**	2	0	15.88 (0.76-331.23)	0.016	BIALLELIC, autosomal or pseudoautosomal
5:147357920-147480692 (51%)	SPINK5	0	1	1.06 (0.043-25.92)	0.974	BIALLELIC, autosomal or pseudoautosomal
6:31360255-31453705 (100%)	MICA	7	10	2.23 (0.85-5.89)	0.096	Unknown
7:150183718-150442413 (100%)	GIMAP6	0	1	1.06 (0.04-25.92)	0.974	BIALLELIC, autosomal or pseudoautosomal
7:150183718-150442413 (100%)	GIMAP5	0	1	1.06 (0.04-25.92)	0.974	BIALLELIC, autosomal or pseudoautosomal
8:100106258-100295634 (22%)	VPS13B	0	3	0.45 (0.02-8.75)	0.589	BIALLELIC, autosomal or pseudoautosomal
9:98664812-98718673 (38%)	ERCC6L2	1	0	9.51 (0.39-233.80)	0.091	BIALLELIC, autosomal or

						pseudoautosomal
10:14983323- 15060883 (26%)	DCLRE1C	1	2	1.58 (0.14-17.50)	0.705	BIALLELIC, autosomal or pseudoautosomal
14:45614097- 45671944 (86%)	FANCM	1	0	9.51 (0.39-233.80)	0.091	BIALLELIC, autosomal or pseudoautosomal
15:51041909-51153142 (28%)	SPPL2A	1	0	9.51 (0.39-233.80)	0.091	BIALLELIC, autosomal or pseudoautosomal
15:55480896-5586070 (83%)	RAB27A	0	1	1.06 (0.04-25.92)	0.974	BIALLELIC, autosomal or pseudoautosomal
16:28832565-29044745 (100%)	CD19	1	1	3.17 (0.20-50.74)	0.389	BIALLELIC, autosomal or pseudoautosomal
16:28832565-29044745 (100%)	LAT	1	1	3.17 (0.20-50.74)	0.389	BIALLELIC, autosomal or pseudoautosomal
16:29627349-30199713 (88%)	CORO1A	0	3	0.45 (0.02-8.75)	0.589	BIALLELIC, autosomal or pseudoautosomal
16:85879197-85930400 (0%)<br>2.4 Kb upstream	IRF8	1	0	9.51 (0.39-233.80)	0.091	BOTH monoallelic and biallelic, autosomal or pseudoautosomal
20:22930037-23050806 (100%)	THBD	1	0	9.51 (0.39-233.80)	0.091	MONOALLELIC, autosomal or pseudoautosomal, imprinted status unknown
22:18877787-19007688 (7%)	ISCA-37433-Loss	7	21	1.06 (0.45-2.50)	0.902	MONOALLELIC, autosomal or pseudoautosomal, imprinted status unknown
22:18877787-19007688 (4%)	ISCA-37446-Loss	8	21	1.21 (0.53-2.74)	0.650	MONOALLELIC,

						autosomal or pseudoautosomal, imprinted status unknown

(*) OR was estimated by applying Pagano & Gauvreau 2X2 table correction for 0 values.
(**) One of these two deletions showed no clear boundaries after visual inspection (using Log R ratio plot). Borders were clarified by using the CytoScan SNP-array. This deletion includes exon 3 and 4 with the boundary in or near exon 5 (chr4:151842384-151905670), and is potentially an LoF deletion.
