# Supplementary material for: Rare copy number variation in autoimmune Addison’s disease
Source: Front Immunol. 2024 Mar 18;15:1374499. doi: 10.3389/fimmu.2024.1374499 (PMC10982488; doi:10.3389/fimmu.2024.1374499)
Supplement: Supplementary file 9 [file Table_7.pdf]

**Supplementary Table 7**. Age at diagnosis and comorbidity profile between AAD large rare deletions carries and AAD without large rare deletions.

| Variable | AAD patients **without** Large rare deletions [1169] | AAD Patients **with** Large rare deletions [13] | p-value* |
|---|---|---|---|
| Age: Median<br>(IQR)<br>(Min–Max Range) | 33<br>(23-45)<br>[0-82] | 30.50 (24.75-41.25)<br>[12-50] | 0.77 |
| Sex (male:female) | 452:717 | 1:12 | 0.02 |
| HOT (positive:negative) | 496:652 | 4:8 | 0.57 |
| HTR (positive:negative) | 87:1050 | 3:9 | 0.06 |
| T1D (positive:negative) | 141:998 | 3:9 | 0.18 |
| PA (positive:negative) | 89:1043 | 0:12 | 0.61 |
| VIT (positive:negative) | 103:1034 | 0:12 | 0.62 |
| CLD (positive:negative) | 50:1086 | 0:12 | 1.00 |
| AA (positive:negative) | 25:1110 | 0:12 | 1.00 |
| 17OH (positive:negative) | 92:760 | 1:6 | 0.55 |
| SCC (positive:negative) | 102:941 | 1:10 | 1.00 |

(*) p-value for age median comparison was calculated using Wilcoxon rank sum test or Mann-Whitney test. Other p-values were calculated with Fisher's exact test.

Abbreviations**: HOT**, Hypothyrodism; **HRT**, Hyperthyroidism; **T1D**, Type 1 diabetes; **PA**, Pernicious anemia; **VIT**, Vitiligo; **CLD**, Celiac disease; **AA**, Alopecia areata; **17OH**, 17OH autoantibodies; **SSC**, SSC autoantibodies.
