# Supplementary material for: Rare copy number variation in autoimmune Addison’s disease
Source: Front Immunol. 2024 Mar 18;15:1374499. doi: 10.3389/fimmu.2024.1374499 (PMC10982488; doi:10.3389/fimmu.2024.1374499)
Supplement: Supplementary file 10 [file Table_8.pdf]

Supplementary Table 8. Rare CNV frequency distribution in patients negative for 21-OH autoantibodies

(a) Patients negative for 21-hydroxylase autoantibody (21-OH-) vs. Controls

CNV type	Counts		Frequency		Association	
	CNVs 21-OH- [n = 201]	CNVs Controls [n = 3,810]	Cases	Controls	OR (95% CI)	*P*
DELs	140	2615	0.70	0.69	1.05 (0.77-1.43)	0.76
DUPs	124	2367	0.62	0.62	0.98 (0.73-1.31)	0.90

(b) Patients positive for 21-hydroxylase (21-OH+) vs. patients negative for 21-hydroxylase (21-OH-)

CNV type	Counts		Frequency		Association	
	CNVs 21-OH+ [n = 1,182]	CNVs 21-OH- [n = 201]	Cases	Controls	OR (95% CI)	*P*
DELs	827	140	0.70	0.70	1.02 (0.73-1.41)	0.93
DUPs	721	124	0.61	0.62	0.97 (0.71-1.32)	0.85

(c) Distribution by interval size in patients negative for 21-hydroxylase (21-OH-) vs. controls

CNV type	CNV length	Counts		Frequency		Association	
		CNVs 21-OH- [201]	CNVs Controls [3,810]	Cases	Controls	OR (95% CI)	*P*
DELs	50KB_100KB	79	1298	0.40	0.34	1.25 (0.94-1.68)	0.14
	100KB_200KB	43	919	0.21	0.24	0.86 (0.61-1.21)	0.39
	200KB_500KB	14	323	0.07	0.09	0.81 (0.47-1.41)	0.45
	500KB_1000KB	4	65	0.02	0.02	1.17 (0.42-3.24)	0.76
	> 1000KB	0	10	0.00	0.003	0.90 (0.05-15.38)	(*)0.94
DUPs	50KB_100KB	48	1050	0.239	0.276	0.83 (0.60-1.15)	0.25
	100KB_200KB	40	614	0.199	0.161	1.29 (0.91-1.85)	0.16
	200KB_500KB	27	488	0.134	0.128	1.06 (0.70-1.60)	0.80
	500KB_1000KB	6	150	0.030	0.039	0.75 (0.33-1.72)	0.50
	> 1000KB	3	65	0.015	0.017	0.87 (0.27-2.80)	0.82

(*) OR was estimated by applying Pagano & Gauvreau 2X2 table correction for 0 values.
